# Tuberculosis (Biographies of Disease)

**DOI:** 10.3201/eid1609.100925

**Published:** 2010-09

**Authors:** Rachel Albalak

**Affiliations:** Author affiliation: Centers for Disease Control and Prevention, Atlanta, Georgia, USA

**Keywords:** Tuberculosis, tuberculosis and other mycobacteria, disease, bacteria, book review

A great story has drama, mystery, conflict, interesting characters, intrigue, and excitement. The story of tuberculosis (TB) has all of these. It is a story of epic proportions. Carol Dyer’s Tuberculosis (Biographies of Disease) is not a textbook or a history book; it tells us the story of this fascinating but deadly disease.

Dyer, a science writer, takes us from the mummies of ancient Egypt, with their visible signs of TB, to the skepticism that met Robert Koch’s discovery of the tubercle bacillus, to today’s global TB public health emergency. Thought by many to be a disease of the past, the final chapters of the book jolt us into today’s reality.

As the drama unfolds, Dyer describes how TB ravaged Europe’s working class during the industrial revolution. More personal accounts from the Romantic Age are especially interesting. She tells us how 6 siblings of the famed literary Bronte family died of TB. She describes the tragic death from TB of the poet John Keats at age 26. And the personal anecdotes continue; from the scientists who advanced our knowledge of TB to the artists whose lives and art were affected. By describing its influence on literature and the arts, Dyer brings to life the profound effects of TB on humanity.

Her discussion of the victories and setbacks in the fight against TB provide a context for what she considers to be the story’s main plot: how is it possible that TB remains a leading cause of death from infectious disease globally? Her description of the complex biology of the organism and the societal characteristics of the disease help us understand why, despite the discovery of effective chemotherapy, TB continues to devastate.

The final chapters provide a sobering picture of the current state of TB. Dyer describes the serious effect of the HIV epidemic on TB and warns of the alarming rates of more dangerous forms of drug-resistant TB. However, the story she tells ultimately becomes a hopeful one. She discusses how the world community has come together, leading to new funding initiatives and prevention and control strategies.

This book is a quick and easy read (120 pages). However, the organization of its 8 chapters is peculiar. The reader might be tempted to skip to the third chapter on the history of TB or even stop reading, as he or she get bogged down in some of the medical details in the first 2 chapters. Occasional overuse of technical detail gets in the way of the story. Sidebars with anecdotes and scientific summaries, scattered throughout, are a nice addition to the format of the book. The timeline at the end is also helpful.

The book falls short in describing the epidemiology of TB. More quantitative information would provide the reader with a better understanding of the magnitude of the problem. Despite this issue and several minor technical inaccuracies, the book is informative and at times exciting. It captures all the elements of this great story. Overall, this book is a great read for public health professionals and the general public. For the reader engaged in global public health efforts, the book should be a call to action.

